# Serum Insulin-like Growth Factor-II Is Associated with Poor Poststroke Outcomes in Males: A Secondary Analysis

**DOI:** 10.3390/ijms26125525

**Published:** 2025-06-09

**Authors:** Christian Glamheden, N. David Åberg, Gustaf Gadd, Daniel Åberg

**Affiliations:** 1 Department of Internal Medicine and Clinical Nutrition, Institute of Medicine, The Sahlgrenska Academy at University of Gothenburg, 41390 Gothenburg, Sweden; chgl98@gmail.com (C.G.); david.aberg@medic.gu.se (N.D.Å.); gustaf.gadd@vgregion.se (G.G.); 2 Region Västra Götaland, Department of Acute Medicine and Geriatrics, Sahlgrenska University Hospital, 41345 Gothenburg, Sweden; 3 Region Västra Götaland, Department of Specialist Medicine, Sahlgrenska University Hospital, 41345 Gothenburg, Sweden

**Keywords:** insulin-like growth factor-II, sex, functional outcome, mortality, stroke severity

## Abstract

The insulin-like growth factor (IGF) system has significance for poststroke outcomes. Previously, we reported that low serum IGF-II (s-IGF-II) in the acute phase is associated with poststroke mortality, and that IGF-II is lower among males. Given the known interactions of the IGF system and estrogen receptor signaling, s-IGF-II may have sex-specific effects. In this study, we conducted a secondary analysis of sex differences in s-IGF-II and poststroke functional outcomes and mortality after ischemic stroke (IS) in the Sahlgrenska Academy Study on Ischemic Stroke (SAHLSIS, males; *n* = 315, females; *n* = 177). Functional outcomes were assessed using the modified Rankin scale (mRS) at 3 months and 2 years poststroke. Survival was recorded for 7 years or until death. Males in the lowest quintile of acute s-IGF-II had a higher poststroke mortality, with a crude hazard ratio [HR] of 2.52 (95% confidence interval [CI]) 1.59–3.99) and an adjusted HR of 1.83 (95% CI 1.09–3.06). No corresponding significant association was observed in females. Although acute s-IGF-II was crudely associated with poor functional outcomes among males after 3 months and 2 years, these associations were not independent of initial stroke severity in adjusted models. In conclusion, low levels of acute s-IGF-II are linked with poststroke mortality among males, but not significantly in females. Further studies are, however, warranted with sex hormone analysis, consideration of specific cause of death, and more females.

## 1. Introduction

### The Insulin-like Growth System

Insulin-like growth factor-I (IGF-I) and insulin-like growth factor-II (IGF-II) are mitogenic hormones. The insulin-like growth factors are, to some extent, produced in most tissues, though mainly in the liver [1]. They are single-chain polypeptides with 43% (for IGF-I) and 41% (for IGF-II) homology to insulin [2]. Their bioavailability is regulated by IGF-binding proteins (IGFBPs), mainly via IGFBP-2 in the brain and via IGFBP-3 outside the central nervous system (CNS). IGF-I secretion is largely dependent on growth hormone (GH), whereas IGF-II is influenced by GH to a much lesser degree [3,4].

IGF-I and IGF-II are required in the CNS for normal fetal brain development, whereas the role of IGF-II in adults remained less clear until ten years ago [5]. For IGF-II, there are still considerably fewer reports regarding brain effects, especially from a clinical perspective. Like IGF-I, IGF-II passes through the blood–brain barrier [1]. In addition to being expressed locally within the CNS, IGF-II binds to both the IGF receptor I and the IGF receptor II, albeit with a higher affinity to the IGF-receptor II [2,6]. Further, the IGF-I and -II receptors are abundantly and widely expressed in non-neuronal structures, such as vascular walls and the choroid plexus [2], where angiogenesis is known to be a key factor in stroke recovery (for a review, see [7]).

Components of the IGF system are indeed involved in the risk of IS and poststroke functional outcomes. A Danish case–control study comprising 57,503 participants with 254 IS cases during a 3.1-year follow-up showed that the lowest quartiles of s-IGF-I and s-IGF-II were associated with approximately twofold and non-significant 1.4-fold higher adjusted risks for stroke, respectively [8]. IGF-II is likely involved in the inflammatory response after ischemia, as an experimental study showed an increased expression of IGF-II mRNA in activated macrophages localized to the infarct zone in the rat brain [9]. Similarly, IGF-II immunoreactivity increased 10 days after hypoxia, mainly in the cortex and microglia [10].

Regarding sex differences in stroke epidemiology, it is known that males have a generally higher stroke incidence and poststroke mortality than females, although, with advanced age, this difference fades [11].

Since IGF-II partially binds the IGF-I receptor [2,9] and there is a link to estrogen receptor signaling [12], the link between IGF-II and sex-specific morbidity is of interest. Indeed, the menopausal decline in systemic estrogen has been suggested to explain the post-menopausal increased risk of neurodegenerative diseases in females [8,13], whereas, to our knowledge, the sex-specific effects of poststroke IGF-I and IGF-II have not been reported or studied. We recently published results indicating that low serum IGF-II (s-IGF-II) in the acute phase of ischemic stroke is associated with mortality after ischemic stroke [14], where we noted a small but significantly higher level of s-IGF-II in males. Thus, there is a rationale to investigate whether there is a sex-specific difference in stroke recovery regarding s-IGF-II, especially at a less advanced age. The hypothesis was, therefore, that low s-IGF-II in males, with virtually no serum estrogen, may worsen the risk of poor poststroke and mortality outcomes more than in females in our relatively young SAHLSIS cohort [15].

## 2. Results

### 2.1. Baseline Characteristics

Of the 600 patients included, there were 492 IS cases with IGF-II data available, comprising 315 males and 177 females. Their clinical characteristics are presented in Table 1. The observed mean age was 57 years. The levels of LDL and BMI were almost the same and had no significant differences, while the levels or frequencies of hypertension, diabetes, smoking, insulin resistance, and high-sensitivity CRP were higher among the cases than the controls (Table 1A).

Correlation analysis showed that acute IGF-II was correlated significantly with age (r = −0.16, *p* < 0.001), NIHSS (r = −0.12, *p* < 0.05), hs-CRP (r = −0.17, *p* < 0.001), and LDL (r = 0.11, *p* < 0.01), but not significantly with BMI, hypertension, insulin resistance, smoking, or diabetes. When analyzing males separately, the correlation between IGF-II and age was stronger (r = −0.19, *p* < 0.001), as was that with the presence of diabetes (r = −0.11, *p* < 0.05), while hypertension, smoking, and BMI remained statistically non-significant.

### 2.2. S-IGF-II and Sex Difference

As reported earlier [14], males with IS had statistically significantly lower s-IGF-II in the acute (9.7%) and 3-month (10.4%) poststroke phases compared with females (Table 1B), although age and other proportions were not statistically different. It can be noted that there were more male stroke patients than females (*n* = 315 vs. *n* = 177, Table 1B), as expected in this age cohort (below 70 years). For comparison, also in the controls, s-IGF-II was 7.3% lower among males than females (*p* < 0.001). Further, when comparing patients and controls, there was a pattern of females having a higher poststroke augmentation of IGF-II than males. Specifically, females had a relatively higher elevation of acute IGF-II than males (4.9%, *p* < 0.001 vs. 2.2%, *p* = 0.1) with respect to the healthy controls. Regarding the rest of the parameters, there were no significant differences, but a tendency (*p* = 0.055) towards more smoking was observed among female patients (45% in females vs. 36% in males, Table 1B). In males, there was a small correlation between acute IGF-II and poor functional outcomes after 3 months (r = −0.12, *p* < 0.05) and 2 years (r = −0.12, *p* < 0.05), as well as a correlation with all-cause mortality (r = −0.20, *p* < 0.01), whilst in women, acute IGF-II was correlated neither with functional outcomes nor all-cause mortality.

### 2.3. Sex Differences in IGF-II and Mortality

**Figure 1:**
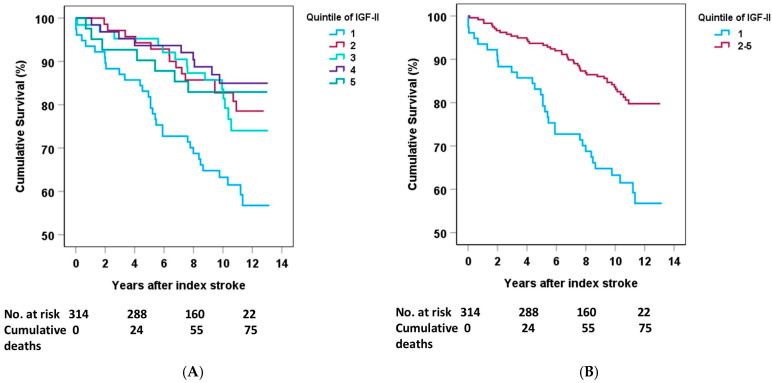
S-IGF-II in the acute phase after stroke among males and its association with overall poststroke mortality. Low IGF-II in the acute phase after ischemic stroke is associated with an increased risk of overall mortality. (**A**) Shows the individual quintiles (for the legend, see the inset), while Figure 1B compares the lowest with the 2nd–5th merged into one group. The lowest quintile is marked with light blue in both figures; in (**B**), the merged group is marked with purple. Log-rank test in (**A**): *p* = 0.006 quintile 1 vs. 2, *p* = 0.022 quintile 1 vs. 3, *p* = 0.001 quintile 1 vs. 4, *p* = 0.018 quintile 1 vs. 5. Log-rank test for overall comparison in (**A**): *p* < 0.001; in (**B**): *p* < 0.001. Numbers at risk are presented as after the year of index stroke (4, 8, and 12 years post-stroke).

The follow-up time was 7 years or until death, with a median of 10.6 years (maximum 13.2 years), as previously reported [14]. The Kaplan–Meier survival curves for males revealed an increased mortality rate in the lowest quintile of acute IGF-II compared with the other quintiles (Figure 1A). Comparisons of any of quintiles 2–5 vs. 1 were all statistically significant, as can be graphically discerned in Figure 1A. The similar mortality rates for quintiles 2–5 justified merging these into one group and subsequently comparing this group with the lowest quintile 1 [14]. Thus, comparing the lowest quintile 1 with quintiles 2–5, the association with cumulative deaths after stroke was also significant (Figure 1B). The association between acute IGF-II and mortality in males was also significant in the crude model of the Cox regression (quintile 1 vs. quintiles 2–5: HR 2.52, 95% CI 1.59–3.99). Table 2 shows the results after adjustment for the covariates in Model A (quintile 1 vs. quintiles 2–5: HR 2.10, 95% Cl: 1.32–3.26), Model B (quintile 1 vs. quintiles 2–5: HR 1.92, 95% Cl: 1.18–3.17), and the fully adjusted Model C (HR 1.83, 95% Cl: 1.09–3.06). When adjusting for BMI separately, there was no change in the results regarding IGF-II and mortality.

**Figure 2:**
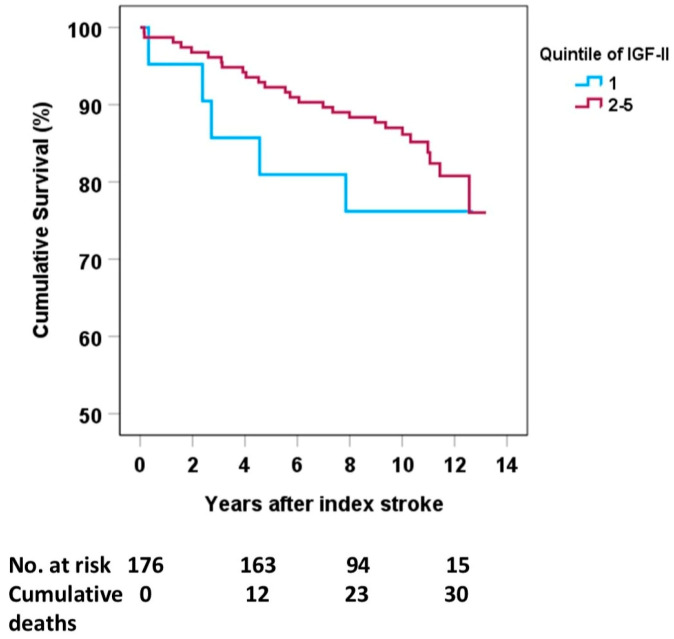
S-IGF-II in the acute phase after stroke and overall poststroke mortality among females. The light blue line is the lowest quintile 1, and the purple line is the merged group of the 2nd–5th quintiles. Log-rank test: *p* = 0.135. Numbers at risk are presented after the year of index stroke (4, 8, and 12 years post-stroke).

The crude association between acute quintile 1 vs. quintiles 2–5 of IGF-II and mortality in females was non-significant (HR 1.58, 95% CI 0.61–4.12, Supplementary Table S1), and remained non-significant after further analyses with adjustments of covariates. When comparing the lowest quintile 1 with the merged quintiles 2–5 among females (Figure 2) or the lowest quintile 1 with any other quintiles 2–5, the log-rank test was non-significant.

IGF-II after 3 months was not related to long-term mortality in either males or females.

As smoking was borderline significantly higher in females than males (Table 1B), we carried out an additional specific sensitivity analysis apart from Model B in the regressions, which included smoking among other cardiovascular covariates. First, we added only smoking in the regression models. As compared with the crude model, this changed the 7-year HR for mortality very little in males (HR 2.54, 95% CI 1.59–4.05) and in females (HR 1.71, 95% CI 0.64–4.52), thus marginally attenuating the sex difference.

Furthermore, as s-IGF-II was slightly higher in females, we performed a sensitivity analysis with sex-specific boundaries of s-IGF-II quintiles regarding regressions for 7-year mortality. These analyses showed similar patterns of significant associations for the s-IGF-II quintiles in males, contrasting with the non-significant associations for females.

### 2.4. Sex Differences in IGF-II and Functional Outcomes

The binary logistic regression analyses showed an association between acute IGF-II and poor functional outcomes (mRS 3–6) for males (Table 3). Of note are the crude associations between acute quintile 1 vs. quintiles 2–5 of IGF-II and poor functional outcomes; these were significant for both the 3-month and 2-year crude ORs for mRS 3–6 (2.72 [95% Cl 1.51–4.92] and 2.59 [Cl 1.44–4.66], respectively). The association abided both 3 months and 2 years after stroke, with significant ORs after adjustment for age and cardiovascular risk factors (3-month mRS 3–6: model B, OR 2.22, 95% Cl 1.17–4.68, 2 years: model B OR 2.00, 95% Cl 1.06–3.79, Table 3). However, the associations were not independent of initial stroke severity, as statistical significance was lost after adjustment for stroke severity (Model C, Table 3). For women, the corresponding OR was non-significant in the crude models of 3 months (mRS 3–6 OR 1.35, 95% Cl 0.46–4.02, *p* = 0.59) and 2 years (OR 0.84, 95% Cl 0.27–2.68, *p* = 0.77 (Supplementary Table S2)) and remained non-significant after further adjustments (Supplementary Table S2).

## 3. Discussion

### 3.1. Summary

This study aimed to investigate poststroke s-IGF-II in males and females and whether this influenced functional outcomes and mortality. In a previous study [14], the data supported the idea that IGF-II is a biomarker for IS and that the lowest quintile is associated with an increased mortality risk. In retrospect, we noted that s-IGF-II differed in males and females, leading to this secondary analysis. Accordingly, the present study showed that the lowest quintile of IGF-II among males, but not females, has a prominent association with mortality. Furthermore, only in males was the association between IGF-II in the acute phase and mortality independent of all covariates.

### 3.2. Sex-Specific Neurological Outcomes of IGF-II

While some studies have suggested that a high level of s-IGF-I is associated with better poststroke neurological outcomes [16,17], our previous data regarding s-IGF-II and poststroke functional outcomes were the first in this context regarding s-IGF-II [14]. Specifically, we reported that a low level of acute s-IGF-II was associated with poor functional outcomes after 3 months and 2 years, although the associations did not withstand adjustments for covariates. Similarly, in the present secondary study, the significant association between IGF-II and functional outcomes in males (Models A and B, Table 3) lost significance after adjustment for stroke severity (Model C). Thus, although the difference between sexes in acute-phase s-IGF-II appears to have some implications for poststroke functional outcomes, it is undoubtedly attenuated by age and cardiovascular covariates (Models A and B) and fades into small, non-significant ORs after adjustment for stroke severity (Model C; Table 3). This is not surprising, as stroke severity is a factor closely related to the outcome after ischemic stroke, both mechanistically and in terms of morbidity, such as with poor functional outcomes (for a review, see [18]). In our study, stroke severity was correlated with poor functional outcomes after three months (r = 0.67) and after 2 years (r = 0.50), both *p* < 0.001, whereas all-cause mortality was closely correlated with stroke severity (r = 0.12, *p* < 0.05). Adding stroke severity to our model (Model C) abolished the association regarding functional outcomes, whereas all-cause mortality remained significant in Model C. This may have been because mortality is not only linked to acute stroke morbidity, but also to other long-term cardiovascular morbidity and other causes of death.

### 3.3. Other Sex-Specific Effects in Stroke

Apart from sex-specific differences in poststroke s-IGF-II, there are several other known sex differences in stroke epidemiology. For example, ischemic stroke is more prevalent among males [19], hypertension is more common among males [20], and males tend to have their strokes at an earlier age than females [19,20]. Could our findings regarding the difference in s-IGF-II between the sexes be explained by these confounders? Besides the difference in s-IGF-II, smoking was the only baseline parameter with a tendency towards a difference between the sexes (Table 1, *p* = 0.055). This trend of smoking differences is in line with the present epidemiology in Sweden, where smoking is more common among females, especially at a younger age [21]. Nevertheless, smoking was included in our adjustments, and it did not correlate significantly with s-IGF-II. There were also no significant correlations between s-IGF-II and hypertension or BMI. Taken together, it appears that age may be the most relevant confounder. There are several reasons for this. First, females tend to have their first stroke at an older age [20]. As the SAHLSIS study only included participants below 70 years of age, it may be speculated that the sex difference could have disappeared if older patients had been included. However, there were no significant age differences between the sexes in the present study (males 57.7 years vs. females 56.3 years). This is speculative but possible given that sex differences in stroke incidence disappear at advanced ages above 85 years [11]. Second, in Model A, which adjusted for age in comparison with the crude association, we observed a relative attenuation of about 10–40% for different outcomes in males, which was much greater than that for Model B vs. Model A. Third, although small in effect size, we also found a correlation between age and IGF-II (r = −0.16). However, since we included adjustments for age, smoking, hypertension, diabetes, and stroke severity in the regression analyses, and significant associations remained, they cannot fully explain our findings.

Other residual confounders that could explain our findings are specific levels of sex hormones. In females, it is, for example, known that the incidence of stroke increases markedly after menopause [20] in comparison with the more even increase in males. Unfortunately, the SAHLSIS study did not measure estradiol, androgens, or testosterone in the blood, requiring larger serum volumes than those used for peptide hormone analysis. A prospective study regarding sex hormones and IS showed that an extremely low testosterone concentration was associated with a high risk of IS in males [22]. In another report, the specific interactions between the IGF system and sex hormones were reported; unfortunately, no follow-up outcomes were reported [23]. Specifically, the strongest correlation was between sex-hormone-binding globulin (SHBG) and IGF-II in males (r = −0.41, *p* < 0.001). Still, there was a correlation between IGF-II and SHBG in females, but it was weaker (r = −0.24). Synthesizing these reports, it may be speculated that males with a higher risk of IS in the lowest quintile also had lower concentrations of testosterone. However, most of the association between low levels of testosterone and a high risk of IS was mediated by the two covariates, hypertension and BMI [22]. We adjusted for hypertension and used additional models for BMI in this study, with minimal impacts on the associations between IGF-II and mortality. Thus, it appears that while BMI and hypertension did not mediate our associations, SHBG could be a mediator. This needs further exploration.

As s-IGF-II is higher in females, we performed a sensitivity analysis with sex-specific boundaries of quintiles. However, for all regressions, the same pattern of significant associations for s-IGF-II quintiles in males was retained, contrasting with non-significant associations for females. This is in line with the crude correlations shown above.

### 3.4. Potential Biological Pathways of IGF-II Action

There are several conceivable ways by which s-IGF-II may exert its effects. As previously stated, IGF-II seems to be neuroprotective, and it activates macrophages’ immune response in the infarct zone [9]. This is linked to increased phagocytosis and relieving post-stroke metabolic requirements, possibly ameliorating stroke recovery [9]. Another mechanism by which IGF-II might act is by enhancing cell genesis—specifically, angiogenesis and neurogenesis—during the recovery phase. Although this has been thoroughly reported for IGF-I (for reviews, see [24,25]), to our knowledge, this has only been investigated for IGF-II treatments without experimental stroke [26]. In addition, a recent cross-sectional study on IS reported that a low expression of IGF-II (quartile 1) mRNA in peripheral plasma exosomes was related to stroke risk, and IGF-II was suggested as a clinical biomarker [27]. In this study, IGF-II was increased among stroke patients compared with controls, and although IGF-II was not independently associated with functional outcomes, our findings suggest the idea that when the IGF system fails to reach a threshold of IGF-II, the recovery of the infarcted zone becomes worse. Several methods of modulating the IGF system have been suggested. The serum levels of IGF-I can be increased with the intravenous administration of tissue plasminogen activator [28], and today, recombinant IGF-I can be given to individuals with a short stature when IGF deficiency is present [29]. Recombinant IGF-II is only available for experimental studies, but there can be other ways to modulate IGF-II activity. For example, the small protein RNA-binding motif protein 3 was upregulated in hypothermia and promoted IGF-II expression and secretion in the subgranular zone of the adult rodent dentate gyrus [30]. In a previous study [16] where IGF-II was analyzed according to the different TOAST subtypes, the cardioembolic etiology had lower IGF-II values. In this study, we found somewhat lower s-IGF-II values among males. Therefore, it could be speculated that males with cardioembolic stroke could be the target group with the highest benefit. Although we have data on TOAST, we lack the power to execute sex-specific analysis regarding whether IGF-II is associated with outcomes in the different ischemic stroke subgroups in our study. Nevertheless, we analyzed a group of cardioembolic strokes (*n* = 56 in males and *n* = 25 in females) and observed that the hazard ratios did not change much (unadjusted HR = 1.77 for males and HR 0.55 for females), with the expected wide, non-significant confidence intervals. Thus, although IGF-II appears to be a possible predictive factor for mortality after stroke, our data are only hypothesis-generating and must be confirmed in future repeated studies.

### 3.5. Strengths and Limitations

The SAHLSIS study has its strengths and limitations when analyzing IGF-II. It is a relatively large cohort study with controls, and the present investigation primarily used patients with IS and successful IGF-II analysis (*n* = 492). One of the strengths of SAHLSIS is its long-term follow-up with a median of 10.6 years, which provides relatively many endpoints for survival analysis. A limitation is that the male group (*n* = 315) was significantly larger than the female group (*n* = 177). This reflects the fact that ischemic stroke is more common among males than females in populations below 70 years of age. Unfortunately, the fact that there were fewer females in SAHLSIS resulted in a smaller number of events among females during follow-up. Consequently, there is less statistical power, especially in women, increasing the risk of statistical errors. With a greater statistical power, the non-significant point estimate HR of 1.58 for increased 7-year mortality in females could have emerged as significant, but would likely still be less robust than in males. In addition, having less statistical power makes the analysis particularly susceptible to adjustments in regression models. Specifically, according to the statistical models of Peduzzi et al. [31], approximately 10 events per variable are necessary to retain statistical robustness in regression models. For the present report’s binary regression models, which are shown in Table 3, the groups with poor outcomes were sufficiently robust for males (poor outcome; *n* = 64) in Models A–C. In females (Supplementary Table S2), the groups were sufficient for Model A, but they were too small for proper power in Models B and C (poor outcome; *n* = 38). In the Cox regression models for males, which are shown in Table 2, statistical robustness was retained for 3–4 variables; hence, Model C has a limited robustness. In the Cox regression for females (Supplementary Table S1), we lacked enough events (*n* = 5) to perform reliable adjustments for covariates. Therefore, the ability to adjust for confounders was generally limited in this study and posed an increased risk of not having adjusted for residual confounders. As smoking frequency was borderline significantly higher in females than in males (45% vs. 36%, *p* = 0.055), we performed a specific sensitivity analysis for this covariate, which resulted in very wide confidence intervals but had no apparent effect on the sex difference in the associations. Still, both smoking and unknown residual confounding are possible. To draw safer conclusions, especially for females, additional case recruitment would be appropriate. Furthermore, the inclusion of stroke patients and controls of predominantly northern European descent may restrict the results’ generalizability to other ethnic groups. In addition, a limitation is that the controls did not represent the population in terms of cardiovascular disease, since the study excluded controls with previous cardiovascular or cerebrovascular events. On the other hand, the control group was only used for s-IGF-II reference and had no outcome analysis. Furthermore, the study was initiated in an era before the wide use of thrombolysis and thrombectomy, though thrombectomy is now routinely used with other antiplatelet and anticoagulant regimes. However, this is also an advantage, as poststroke growth factors may be affected by interventions. Thus, the present study may represent a more accurate description of a naive IS event course.

The effect of low s-IGF-II in males was more robust for mortality, and it withstood all adjustment models. Although controversial, this may have a link to reports of associations between s-IGF-II and cancer mortality. Indeed, high s-IGF-II has been reported to be correlated with cancer risk, especially colorectal, breast, prostate, lung, and colon cancer [32], and low s-IGF-II has been correlated with colorectal cancer mortality [33]. Our mortality data were retrieved and calculated based on any cause of mortality, and we did not have full access regarding the specific cause of death, which limited the conclusions that we could draw from our mortality data. Since malignancy is the second-most common cause of death in Sweden, this might have affected our results regarding mortality. However, this is probably of minor concern in the present study, which is mostly because death due to CVD is the predominant cause of death after stroke [34].

## 4. Materials and Methods

### 4.1. Study Population

In 1998, the Sahlgrenska Academy Study on Ischemic Stroke (SAHLSIS), a prospective case–control observational study of ischemic stroke in adults of northern European descent under 70 years of age, was initiated [15]. The present investigation involves a secondary analysis of the results regarding s-IGF-II [16]. In SAHLSIS, patients with acute ischemic stroke (*n* = 600) were consecutively recruited until 2003 from four stroke units in western Sweden [15]. Healthy controls (*n* = 600) were randomly selected from participants in a population-based health survey from Gothenburg or the Swedish Population Register and matched for age, sex, and geographical residence area, as described in [14]. SAHLSIS was approved by the Ethics Committee of the University of Gothenburg, and the National Computer Data Inspection Board approved the data-handling procedures. All participants gave their written informed consent. Next of kin gave consent for participants who were unable to communicate.

### 4.2. Data Collection

The baseline data for SAHLISIS were collected through a structured questionnaire, clinical evaluation, and venous blood sampling [15]. Smoking was classified as current smoking or not smoking, the latter meaning that the participant was a never-smoker or had ceased smoking a year or more before entering the study. Hypertension was defined as current hypertensive treatment and blood pressure above 160 mmHg systolic or 90 mmHg diastolic. Stroke severity was assessed with the Scandinavian Stroke Scale (SSS) and later recalculated to the now more commonly used National Institutes of Health Stroke Scale (NIHSS) with the algorithm NIHSS = 25.68 − 0.43 × SSS [35].

Functional outcomes were assessed using the modified Rankin Scale (mRS) 3 months and 2 years after the index stroke, as described in [15]. The mRS scale was dichotomized at the threshold of values for independent (mRS 0–2) or dependent (mRS 3–6) functional states.

Blood samples were collected twice for cases in the acute phase (median: 4 days; range: 1–10 days after the index stroke) and after 3 months (median: 101 days; range: 85–125 days after the index stroke), or once for controls. The sampling was conducted between 8:30 and 10:30 AM after an overnight fast for at least 8 h. The serum was isolated within 2 h via centrifugation at 2000× *g* at 4 °C for 20 min and stored at −80 °C before the assay. As reported previously, the level of s-IGF-II was measured using an enzyme-linked immunosorbent assay (ELISA) from Mediagnost, Reutlingen, Germany [14]. The inter-assay coefficient of variation (CV) was 7.0%, and the intra-assay CV was 4.2%. Low-density lipoprotein (LDL) was analyzed using standardized methods at the Department of Clinical Chemistry at the Sahlgrenska University Hospital [14]. Diabetes mellitus (henceforth diabetes) was defined as a fasting plasma glucose of >7.0 mmol/L, a fasting blood glucose of >6.1 mmol/L, or undergoing treatment for diabetes via pharmacological or dietary methods. To calculate HOMA-IR, the following formula for mmol/L was used: fasting insulin (µU/L) × fasting glucose (nmol/L)/22.5 [36]. High-sensitivity CRP (hs-CRP) was analyzed in serum using a solid-phase chemiluminescent immunometric assay on Immulite 2000 (Diagnostic Products Corp, Los Angeles, CA, USA) with the manufacturer’s reagents as directed.

### 4.3. Statistics

We used SPSS version 29 (IBM Corp., Armonk, NY, USA) to perform the statistical analyses. An independent *t*-test was performed to compare means. To attain correlation coefficients, we used Pearson correlation analysis.

Mortality data were collected from the Swedish Cause-of-Death registry. To determine if sex differences in s-IGF-II affected mortality, we first used quintiles for the IGF-II distribution. We then merged the quintiles into two groups, with the lowest quintile 1 being compared with quintiles 2–5, as in a previous study [14]. This finding is also in line with earlier studies on biological and endocrine biomarkers and the IGF family, which suggest an inverse, nonlinear excess risk in deficient subjects, indicating that there may be a threshold level of IGF-II [8,37,38]. The cutoffs for the quintiles of acute s-IGF-II in the total population were as follows: Q1 90.0–611.9, Q2 612.0–698.2, Q3 698.3–768.3, Q4 768.4–861.5, and Q5 861.6–1955 ng/mL, respectively. In the previous study, the lowest quintile was distinguished from the rest when the quintiles were imposed on Kaplan–Meier survival curves, and we used the same comparison in the present investigation. We used Cox regression to calculate hazard ratios and 95% confidence intervals.

To investigate functional outcomes, we used binary logistic regression to calculate odds ratios (ORs) and 95% confidence intervals (CIs) for the risk of poor functional outcomes (i.e., mRS 3–6) after 3 months and 2 years.

To examine the independent effect of s-IGF-II on mortality (Table 2, Supplementary Table S1) and functional outcomes (Table 3, Supplementary Table S2), adjustments were made for the most substantial or probable confounders, as follows: Model A—age, Model B—additionally adjusted for hypertension, diabetes, and smoking, and Model C—additionally adjusted for stroke severity, i.e., the NIHSS score. These five covariates were selected from baseline data to attain a reasonably statistically robust model [31] since the number of patients and events was limited (males; *n* = 315, females; *n* = 177). As presented in our former study of IGF-II in SAHLSIS [14], the number of missing values was low, and no covariates in the present study had a prominent number of missing values. Hence, data imputation was not carried out in this study.

## 5. Conclusions

The data from our study show that acute s-IGF-II levels were lower in males than in female IS patients and that low acute IGF-II levels in males were independently associated with a higher risk of overall mortality after IS, even after adjusting for all models. There was a similar but weaker pattern for poststroke functional outcomes with corresponding crude s-IGF-II associations for low s-IGF-II and poor functional outcomes (mRS) in males. However, these associations were not independent of initial stroke severity, as they were attenuated below significance when stroke severity was added as a covariate. To conclude, there was a difference in effect size for risk with low IGF-II regarding long-term poststroke mortality between males and females. In males, the regressions remained significant after all adjustments; however, in females, the lower risk estimate was not even significant in the unadjusted model. Definite conclusions are, however, hampered by the lower statistical power for females, and whether this difference is a causal factor for poststroke outcomes in IS needs further investigation. Concerning this, additional studies regarding the outcomes of ischemic stroke with a focus on IGF-II, while including data on all significant sex hormones, as well as the causes of mortality, would be of special interest.

## Figures and Tables

**Table 1:** Baseline characteristics in healthy controls and in all cases (**A**) and for males and females in IS cases (**B**).

**A. Healthy Controls and Patients**		
	**Controls (*n* = 514)**	**All IS Cases (*n* = 492)**
Age (years)	57.2 ± 0.44	57.0 ± 0.44
Hypertension (%)	39	62 ***
Diabetes mellitus (%)	6	19 ***
Current smoker (%)	17	39
BMI, kg/m^2^	26.5 ± 0.18	26.7 ± 0.20
Imputed LDL (ng/nL)	3.33 ± 0.0	3.35 ± 0.04
HOMA-IR	2.13 ± 0.12	4.80 ± 0.25
NIHSS score baseline	NA	5.31 ± 0.25
mRS score 3 months	NA	2.34 ± 0.11
mRS score 2 years	NA	1.91 ± 0.08
hsCRP (mg/L)	3.06 ± 0.26	10.81 ± 1.00 ***
s-IGF-II acute (ng/mL)	712.1 (±5.58)	734.7 ±7.08 *
s-IGF-II 3 months (ng/mL)	NA	736.8 ± 6.91 *
**B. IS Cases, Males and Females**		
	**Males (*n* = 315)**	**Females (*n* = 177)**
Age (years)	57.7 ± 0.52	56.3 ± 0.78
Hypertension (%)	64	57
Diabetes mellitus (%)	21	16
Current smoker (%)	36	45 ^a^
BMI, kg/m^2^	26.9 ± 0.21	26.4 ± 0.42
Imputed LDL (ng/nL)	3.29 ± 0.52	3.44 ± 0.07
HOMA-IR	5.10 ± 0.34	4.28 ± 0.31
NIHSS score baseline	5.12 ± 0.31	5.65 ± 0.44
mRS score 3 months	2.23 ± 0.14	2.53 ± 0.21
mRS score 2 years	1.96 ± 0.12	1.81 ± 0.11
hsCRP (mg/L)	11.5 ± 1.4	9.5 ± 1.3
s-IGF-II acute (ng/mL)	707 ± 8.9	783 ± 10.8 ***
s-IGF-II 3 months (ng/mL)	708 ± 8.7	790 ± 10.2 ***

Data are shown as means (±standard error of the mean). NA, not analyzed; LDL, low-density lipoprotein; BMI, body mass index; HOMA-IR, homeostasis model assessment of insulin resistance; hsCRP, high-sensitivity C-reactive protein. Comparisons between columns were made using *t*-tests for continuous parameters and Chi Square tests for proportions. *p*-values are designated with * *p* < 0.05, *** *p* < 0.001, and ^a^ *p* = 0.055.

**Table 2:** Hazard ratios (HRs) and 95% confidence intervals (CIs) for the risk of mortality in the lowest quintile 1 of acute s-IGF-II vs. quintiles 2–5 among males with ischemic stroke.

Stroke (Males)	Quintile 1	Quintile 2–5	*p*-Value
Deaths (*n*)	31	44	
Crude	2.52 (1.59–3.99)	1.0 referent	<0.01
Model A	2.10 (1.32–3.26)	1.0 referent	0.002
Model B	1.92 (1.18–3.17)	1.0 referent	0.009
Model C	1.83 (1.09–3.06)	1.0 referent	0.022

Hazard ratios were calculated using Cox proportional regression. Data are shown as n, HR (95% CI), and *p*-values. Model A: adjustment for age. Model B: age and cardiovascular risk factors (smoking, hypertension, and diabetes). Model C: age, cardiovascular risk factors, and stroke severity.

**Table 3:** Odds ratios (ORs) and 95% confidence intervals (CIs) for the risk of poor functional outcomes after 3 months and 2 years in the lowest quintile 1 of acute s-IGF-II vs. quintiles 2–5 among males with ischemic stroke.

Stroke (Males)	Quintile 1	Quintiles 2–5	*p*-Value	*n*
3-month mRS 3–6				
Crude	2.72 (1.51–4.92)	1.0 referent	0.002	298
Model A	2.54 (1.38–4.68)	1.0 referent	0.003	298
Model B	2.22 (1.17–4.20)	1.0 referent	0.014	293
Model C	1.18 (0.51–2.74)	1.0 referent	0.701	292
2-year mRS 3–6				
Crude	2.59 (1.44–4.66)	1.0 referent	0.001	308
Model A	2.26 (1.23–4.13)	1.0 referent	0.008	308
Model B	2.00 (1.06–3.79)	1.0 referent	0.033	300
Model C	1.34 (0.65–2.78)	1.0 referent	0.432	299

Odds ratios were calculated using binary logistic regression. Data are shown as OR (95% Cl) and *p*-values. Quintile 1 shows the OR with quintiles 2–5 as the referent. Model A is adjusted for age. Model B: age and cardiovascular risk factors (smoking, hypertension, and diabetes). Model C: age, cardiovascular risk factors, and stroke severity.

## Data Availability

The data presented in this study are available from the corresponding author upon reasonable request. The data are not publicly available due to legal restrictions regarding privacy and ethical issues.

## Supplementary Materials

The following supporting information can be downloaded at: https://www.mdpi.com/article/10.3390/ijms26125525/s1.

## Abbreviations

The following abbreviations are used in this manuscript:

BMI	Body Mass Index
IGF-II	Insulin-Like Growth Factor II
IS	Ischemic Stroke
mRS	Modified Rankin Scale
NIHSS	National Institutes of Health Stroke Scale
SSS	Scandinavian Stroke Scale
