# Supplementary material for: Serum Insulin-like Growth Factor-II Is Associated with Poor Poststroke Outcomes in Males: A Secondary Analysis
Source: Int J Mol Sci. 2025 Jun 9;26(12):5525. doi: 10.3390/ijms26125525 (PMC12192941; doi:10.3390/ijms26125525)
Supplement: Supplementary file 1 [file ijms-26-05525-s001.zip › ijms-3589833-supplementary.pdf]

Supplementary Tables

Supplementary Table S1. Hazard ratios (HR) and 95% confidence intervals (Cls) for the risk of mortality in the lowest quintile of acute s-IGF-II vs the 2nd–5th quintiles among females with ischemic stroke.

Stroke (females)	Quintile 1	Quintile 2-5	p-value
Deaths (n)	5	26	
Crude	1.58 (0.61-4.12)	1.0 referent	0.35
Model A	1.49 (0.57-3.89)	1.0 referent	0.42
Model B	1.42 (0.53-3.82)	1.0 referent	0.49
Model C	1.28 (0.47-3.46)	1.0 referent	0.63

Footnote: Hazard ratios were calculated using Cox proportional regression. Data are shown as n, HR (95% CI), and p-values. Model A: adjustment for age. Model B: age and cardiovascular risk factors (smoking, hypertension, diabetes). Model C: age, cardiovascular risk factors, and stroke severity.

Supplementary Table S2. Odds ratios (OR) and 95% confidence intervals (Cl) for the risk of poor functional outcomes after 3 months and 2 years in the lowest quintile of acute s-IGF-II vs. the 2nd–5th quintile among females with ischemic stroke.

Stroke (females)	Quintile 1	Quintile 2-5	p-value	n
**3-month mRS 3-6**				
Crude	1.35 (0.46-4.02)	1.0 referent	0.59	165
Model A	1.39 (0.46-4.15)	1.0 referent	0.56	165
Model B	1.09 (0.32-3.69)	1.0 referent	0.90	164
Model C	0.31 (0.05-1.92)	1.0 referent	0.21	164
**2-year mRS 3-6**				
Crude	0.84 (0.27-2.68)	1.0 referent	0.77	177
Model A	0.77 (0.24-2.47)	1.0 referent	0.66	177
Model B	0.48 (0.12-1.86)	1.0 referent	0.29	174
Model C	0.20 (0.04-1.06)	1.0 referent	0.20	164

Footnote: Odds Ratios were calculated using binary logistic regression. Data are shown as OR (95 % Cl) and p-values. Quintile 1 shows the OR with quintiles 2-5 is the referent. Model A is adjusted for age. Model B: age and cardiovascular risk factors (smoking, hypertension, and diabetes). Model C: age, cardiovascular risk factors and stroke severity.
